# Dr. Edward Brown-Sequard’s Experimental and Clinical Researches, Epilepsy

**Published:** 1857-06

**Authors:** 


### SELECTIONS.

### *Dr. Edward Brown-Sequard's Experimental and Clinical Researches, applied to Physiology and Pathology.*

EPILEPSY—(Continued).

§ XIII. My experiments upon animals, compared with cases of epilepsy observed in man, throw a great deal of light on what we might call the physiology of epilepsy, that is, upon what concerns the etiology, the seat, and what is vaguely called the nature of this disease. It is easy to show that one or the other of the two series of facts we have to compare, if not both, are in opposition with the various doctrines concerning the production and the seat of epilepsy. A short critical examination of these doctrines will prove the correctness of this assertion.

The time has passed away when men of talent were tempted to place the seat of epilepsy in the pituitary body (Joseph Wenzel), in the pineal gland (Greding), or in the spinal cord (Esquirol Reid). The injuries or organic alterations of these parts, as well as of other parts of the nervous system, may be either the cause or an effect of epilepsy, but none of these parts can be considered as the *essential seat* of this affection. The numerous cases of co-existence of epilepsy and of a disease of the pituitary body, related by Joseph Wenzel (*Beobachtungen ueber den Hirnanhang fallssuchtiger Personen.* Edited by Carl Wenzel, Mainz, 1810), have lost their apparent importance since it has been shown by Romberg (*loco cit.*, p. 685) and others (Rokitansky, Engel and Sieveking, in Handfield Jones's and Sieveking's *Manual of Pathological Anatomy*, 1854, p. 267, *Amer. Ed.*) that the pituitary body may be altered although epilepsy does not exist, and that this neurosis may exist without any apparent alteration in this small organ. There is no part of the nervous centres about which the same argument could not be used.

Many writers have asserted that epilepsy must depend upon a disease of the brain (organic or not), on account of the existence of the cerebral symptoms. It is useless to speak of the authors who have been or who still are unacquainted with the phenomena of reflex actions; I will merely refer for their views to the works of Portal (*loco cit.*, p. 143-155) and Delasiauve (*loco cit.*, p. 27-35). But many physicians of talent, knowing very well what relates to the reflex actions, have considered the brain as the essential seat of epilepsy. Thus, this affection is placed among the so-called cerebral convulsions by very able pathologists, such as Romberg (*loco cit.*), Spiess (*Krankhafte Stor. des Nervensystems* in Wagner's *Handworterbuch der Physiol.*, vol. iii., 2d part, 1846, p. 188), Russell Reynolds (*The Diagnosis of Diseases of the Brain, Spinal Cord, &c.*, 1855, pp. 143 and 174), and others. According to Dr. John Simon, "the intellectual changes which precede, accompany or follow the progress of the disease, its concurrence with insanity, and its tendency to dementia, further mark the convoluted surface of the hemispheres as the primary seat of the morbid process" (*General Pathology, &c.*, 1852, p. 152, *Amer. Ed.*)

The modern physiologists agree in admitting that the brain proper (the cerebral lobes) cannot give rise to convulsions when it is irritated, in animals. Surgeons have sometimes had an opportunity of ascertaining that, in man also, the brain may be cut without producing convulsions. But these facts merely prove that usually the brain proper cannot be excited by our means of excitation. They do not prove that it cannot be irritated by other kinds of irritation. The cerebral lobes, as being the seat of the will, are certainly connected with muscles and can produce contractions in them, as our voluntary movements constantly prove. We think convulsions may result from kinds of irritation (as that of a poison in the blood, for instance) different from those which we usually employ in our experiments. On another side it may be that alterations in the nutrition of the brain, and other causes, produce a change in the vital properties of this organ, or rather give it something that normally it does not possess, viz., the property of causing convulsions when it is irritated. That such a change in the vital powers of the cerebral lobes is possible, we are led to admit, as we know that other parts of the nervous system may acquire vital properties that they have not in their normal state. For instance, some parts of the sympathetic nerve seem to be deprived of sensibility, but inflammation renders them very sensitive; the nerves of tendons seem to be without sensibility, but inflammation renders them evidently sensitive, as has been definitely proved by the well-devised experiments of Prof. Flourens (*Comptes Rendus des Seances de l'Academie des Sciences*, 1856, vol. xliii., p. 639). I might give also as a striking instance of a change in the vital properties of a part of the nervous system, what occurs to the cutaneous ramifications of certain branches of nerves in the face and neck, after an injury to the spinal cord in animals.

I must now say that, although I admit the possibility of the production of convulsions by an irritation of the cerebral lobes, I do not think it is proved that these parts of the nervous system have actually caused convulsions. The facts mentioned by Romberg (*loco cit.*, p. 625–27) do not furnish such a proof. They may be explained by admitting that the convulsions depended upon either an excitation of the sensitive nerves of the meninges, or upon pressure on the parts of the encephalon which are known to be excitable, or upon the disturbance of circulation and nutrition in the excitable parts of the encephalon. The case of a child *Pathol. and Pract. Researches on Diseases of the Brain*, by Abercrombie, 4th Edit., 1845, p. 57), upon whose anterior fontanelle pressure determined convulsions, and the experiments of Portal (*loco cit.*, p. 149), which gave similar results, cannot prove anything, because pressure upon any part of the brain through a small opening in the cranium, acts upon the whole of the encephalon.

Until it is proved that the cerebral lobes have directly caused convulsions, we are not entitled to say that the seat of epilepsy is in them. If it is argued that the brain proper must be the seat of this affection because an idea or a remembrance, or a smell or the sight of certain things, may induce a fit, we answer that these causes of convulsions act in producing an emotion, and that emotions have their seat in the pons varolii and the medulla oblongata, and not in the brain. If it is said that the loss of consciousness implies that the cerebral lobes have something to do with epilepsy, we certainly do not deny it; but what is the relation between these lobes and epileptic fits? How can convulsions, *i. e.*, actions the existence of which imply that a great amount of nervous power is employed —how can they be produced by an organ which has lost its principal function? How can this organ be so active in the production of convulsions just at the time it loses its activity as the organ of volition and perception? How can we admit that an organ assumes actions which it is not known to possess, at the same time that it loses its well-known actions?

Those physicians who maintain that the brain is the primary or essential seat of epilepsy, have too much neglected these difficulties and contradictions. Their only argument consists in saying that it *must* be so because the brain is affected; but we might employ a similar argument to say that many other parts of the nervous centres are the seats of epilepsy because they are evidently affected, and as much as the brain. It is interesting to remark that it is just the same argument that Dr. Marshall Hall employs to shows that the seat of this affection is in what he calls the *true* spinal cord. Such arguments in the end amount to simply an assertion like the following: it is so, not because it is proved to be so, or because the facts agree in allowing us to admit that it is so, but because we cannot explain it otherwise. An argument of this kind is never a decisive one; but it has no value, and ought not to be employed, when the facts are not explained by the hypothesis considered as the only possible explanation, and still more (as is the case with the supposition that the seat of epilepsy is in the brain proper) when there are facts in opposition to the proposed explanation.

We will try to show hereafter that the loss of consciousness in epilepsy may be explained otherwise than by admitting that the brain is the seat of this affection, and that the loss of consciousness, whether it exists alone or with convulsions, may be due to an action beginning elsewhere than in the brain.

As regards the state of the mind and of the senses after an attack of epilepsy, it is not and cannot be a proof that the seat of this disease is in the brain, as those disturbed states may result from various circumstances existing during a fit.

We must now say a few words of a theory which pretends to solve the difficulty above exposed, concerning the coincidence of the loss of action of the brain and of an increased muscular action. The estimable author of a singular but interesting work (*Epilepsy, and other affections of the nervous system which are marked by tremor, convulsion, or spasm, &c.*, 1854), Dr. C. B. Radcliffe, in this book and in his lectures on Epilepsy (*Medical Times and Gazette*, March and April, 1856), grounds an explanation of this difficulty upon the supposed fact that muscular contraction does not depend upon a stimulus by the nervous system, but upon the cessation of all stimulus. Dr. Radcliffe, after Duges and others, thinks that muscular contraction is a purely physical phenomenon, dependent on ordinary molecular attraction when the muscle is *not* stimulated. If the muscles are at rest, it is because an excitation comes upon them, preventing the molecular attraction from producing contraction. If a voluntary movement takes place, it is because the will has suppressed the nervous action which prevented contraction. In a fit of epilepsy, convulsions take place, together with the loss of consciousness, because the brain and other parts of the nervous centres lose their powers at the same time, and stimuli being withdrawn from the muscles, they are left to the action of molecular attraction, and therefore convulsions are produced. It is useless to discuss a theory like this, which is in opposition to almost all the known and the most positive facts of physiology and pathology. I will merely say that if the theory were true, we should always see convulsions in paralyzed muscles, and also after death at the time when nerves lose their power upon muscles.

According to another theory, which certainly deserves much more attention than the preceding, epilepsy depends upon changes taking place in the circulation of blood in the brain proper, and in the other parts of the encephalon. The germ of this theory may be found in the works of many writers, and particularly in the remarkable book of Prichard (*A Treatise on Diseases of the Nervous System*, part 1st, 1822), but Henle has done so much for it that he may be considered as its originator. In his admirable work (*Handbuch der Rationelle Pathologie*, vol. ii., 1st part, 1855, 2d ed., p. 181–3 and p. 403; and 2d part, 1854, p. 46) he tries to show that there are two kinds of epilepsy, one attended with plethora, the other with anæmia. In both there is as a cause of convulsions, a pressure by accumulated blood in the vessels of the base of the encephalon. We may understand easily the congestion of the brain in plethora, but it is not so as regards anæmia. Henle explains it in this last case, in admitting that when anæmia goes on increasing, the blood-vessels of the upper parts of the encephalon becoming empty, the others necessarily become more filled, on account of the impossibility of the cranio-spinal cavity containing less fluid (an impossibility well established by Kellie, Abercrombie, J. Reid and others). As regards the loss of consciousness, it is attributed to an excess of blood pressing upon the brain proper, in plethoric epilepsy, and to the diminution of blood in this organ, in anæmic epilepsy.

Although we think that many thanks are due to Henle for the efforts he has made to show the relations between the phenomena of epilepsy and the state of the blood-vessels of the various parts of the encephalon, we cannot adopt his theory.

In the first place, if a congestion in the two distinct parts of the encephalon (the brain proper and the basis of the encephalon) was sufficient to produce epilepsy, this disease would be much more frequent than it is, and we should not see so often hyperæmia of the encephalon without convulsive fits, and nevertheless powerful enough to cause paralysis, delirium or coma. The great work of Prof. Andral (*Clinique Medicale*, 4th ed., vol. v., 1840, p. 217–292) and almost all the treatises on intermittent fever, and particularly those of Bailly and Maillot, afford decisive proofs of the frequency of cases of encephalic congestion without epilepsy. Henle himself has been obliged to say that *individual disposition* is necessary for the production of this convulsive affection.

In the second place, we object to the theory of the learned German physician, because he gives no proof that the mere mechanical action (pressure) due to accumulated blood in the vessels of the basis of the encephalon, is sufficient to produce convulsions.

In the third place, Henle does not give any clear reason why, in the anæmic epilepsy, the blood-vessels of the brain proper contract, while those of the basis do not; and besides, except concerning lead disease, he does not say what excites contraction in them.

Theories of epilepsy, entirely at variance with the preceding, have been proposed. In the last century, Saillant (*Exper. sur des Animaux pour decouvrir le siege et la cause prochaine de l'epilepsie*, in *Hist. de la Soc. Royale de Medecine*, in 1782 and 1783, p. 88–89), without giving a theory of epilepsy, concluded, from some experiments, that it is easier to cause epileptic seizures in producing alterations in the blood than by irritating the nerves or the brain. Had galvanism been known at the time of the researches of Saillant, and had he employed it to irritate the nervous centres, he would have seen much more violent and lasting convulsions than those he observed after having altered the blood by injections of air, &c. His experiments only show that convulsions may be due to altered blood, a fact well known already before his researches.

One of the most eminent medical writers of our times, Dr. R. B. Todd, has recently proposed a theory of epilepsy, which I must discuss at length, on account of the importance it should have if it were true, and of the value that belongs, necessarily, to any opinion held by such an ingenious and experienced physician.

Dr. Todd says, "I hold that the peculiar features of an epileptic seizure are due to the gradual accumulation of a morbid material in the blood, until it reaches such an amount that it operates upon the brain in, as it were, an explosive manner; in other words, the influence of this morbid matter, when in sufficient quantity, excites a highly polarized state of the brain, or of certain parts of it, and these discharge their nervous power upon certain other parts of the cerebro-spinal centre, in such a way as to give rise to the phenomena of the fit."

Dr. Todd then proceeds to say, that a very analogous effect is observed when strychnine is given to a cold-blooded animal. This drug may be administered in very minute quantities for some time without producing any sensible effect; but when the quantity has accumulated in the system up to a certain point, then the smallest increase of the dose will immediately give rise to the so-well-known peculiar convulsive phenomena, observed in this kind of poisoning. Dr. Todd adds: "This, then, is the humoral theory of epilepsy. It assumes that the essential derangement of health consists in the generation of a morbid matter, which affects the blood, and it supposes that this morbid matter has a special affinity for the brain or for certain parts of it, as the strychnine, in the case just cited, exercises a special affinity for the spinal cord. The source of this morbid matter is probably in the nervous system, it may be in the brain itself. It may owe its origin to a disturbed nutrition—an imperfect secondary assimilation of that organ—and in its turn it will create additional disturbances in the functions and the nutrition of the brain."

"According to the humoral theory, the variety in the nature and severity of the fits depends on the quantity of the poisonous or morbid material, and on the part of the brain which it chiefly or primarily affects. If it affect primarily the hemispheres, and spend itself, as it were, on them alone, you have only the epileptic vertigo. If it affect primarily the region of the quadrigeminal bodies, or if the affection of the hemispheres extend to that region, then you have the epileptic fit fully developed."—(*Medical Times and Gazette*, Aug. 5, 1854, p. 129.)

This theory is nothing but an ingenious hypothesis which Dr. Todd proposes, without trying to prove it. The only reason he adduces to support his theory is, that in the renal epilepsy there is very likely a poison in the blood, but as regards the other kinds of this convulsive affection, he does not say any thing which may lead to the admittance of his hypothesis. Feeling that he had no proof of the correctness of his views, he says: "To give a more definite character to the humoral theory, we need to discover a morbid matter in the blood, in variable proportions, in every case of epilepsy. This desideratum has, as yet, been only partially obtained." Dr. Todd alludes here to the influence of the accumulation of urea in blood, in the cases of renal epilepsy. Leaving aside, for a moment, this kind of epilepsy, we may say against the humoral theory of the eminent British physician: 1st, That we do not know any fact in favor of it; 2d, That there are a great many facts in opposition to it.

Not only we do not know any fact favorable to this theory, but its author seems to be like ourself, in this respect, as he does not relate a single one. We have never read or heard that a poison produced in the brain, has been found in the blood of epileptics, and we cannot imagine on what ground a fact of this kind is considered as probable by the author of the humoral theory or rather hypothesis.

To establish the humoral hypothesis on a solid basis, it would be necessary to show: 1st, That there is always a poison in the blood of all epileptics; 2d, That this poison gradually accumulates in the blood until its quantity has become considerable enough to produce the phenomena of the fit; 3d, That during or after a fit, this quantity diminishes (because if it were not so, the fit would continue or come again and again, after a very short time); 4th, That the nature of the poison varies, so that it acts either on the brain proper alone (producing a mere vertigo,) or on the other parts of the cerebro-spinal centre alone, or on the whole of this centre at once; 5th, That this poison has quite a different influence on the brain proper and on the other parts of the cerebro-spinal centre, destroying the actions of the former and increasing excessively the actions of the latter.

Not only none of these points have been made out, but it seems that no attempt has been made in the way of a demonstration in this respect.

That there is a poison in the blood of epileptics is fact a which, nevertheless, is possible, as there are substances in the blood of every man, healthy or epileptic, which by a transformation or by accumulation, may act as poisons, and be the cause of many of the phenomena of an epileptic seizure; but it is not known whether the quantity or quality of these substances is changed in epileptics, just before the fits.

There are many facts which are in direct opposition with the humoral theory of epilepsy. Certainly it is so for all the cases in which a ligature around a limb or one of its parts, has prevented a fit, and also for the cases in which epilepsy has been cured by the section of a nerve, by an amputation, by the extirpation of a tumor, a tooth or a foreign body, or by the expulsion of calculi, of worms,* &c. If, in all these cases, there was, as the cause of the phenomena of the seizure, a peculiar influence of some poisonous matter on the encephalon, instead of an irritation springing from certain peripheric nerves, the means mentioned would not prevent the fits, and, still less, effect a complete cure of the disease. If we were to admit that it is a poison which causes the phenomena of the seizure in these cases, we should have to admit also that this poison

* A curious case of hystero-epilepsy, due to larvæ in the frontal sinuses, has been recently published by Messrs. Dumenil and Legrand Dussaule. These larvæ, which belonged to five different species, were expelled by the nose, and after their expulsion the patient, who had had violent convulsions for many months, was cured. (See the very useful report on the progress of medicine and surgery, entitled *Annuaire des Sciences Medicales*, par le Dr. Lorain, revu par le Dr. Ch. Robin. Paris. 1856. p. 151.)

acts on the peripheric parts of some nerves, and not on the encephalon. But there is no more ground for this last hypothesis than for the preceding, because the presence of a poison in the blood is a mere supposition. Besides, if this would be a reality instead of a gratuitous supposition, it would remain to be explained why this poison does not act in some way or other after the section of a nerve, or the extirpation of a tooth, &c.

The humoral theory is in opposition with many other facts, among which are those proving that an emotion or various other moral causes may produce a fit of epilepsy. For cases showing, without any doubt, the influence of these causes and their relative frequency, I will refer to the works of Delasiauve (*Loc. cit.*, p. 219–22) and Moreau de Tours (*De l'etoil. de l'epil. et des indications*, &c., in *Mem. de l'Acad. Imper. de Medecine.* 1854. Vol. XVIII. p. 1, *et seq.*)

The facts we have related in § XI., to prove that seizures of epilepsy are sometimes produced by a mere pressure upon or by galvanization of a small part of the skin, are also in direct opposition to the humoral theory. How could a pressure upon the skin produce a fit, every time it is made, if the fits were due only to a peculiar influence of a poison on the encephalon?

The following facts resemble, in many respects, those I have mentioned in § XI., and they also are in complete opposition to the humoral theory: they have been collected by Delasiauve (*loco cit.*, p. 137–38), to show the influence of certain circumstances on some epileptics: 1. A patient could not smell hemp, without having a fit.—(*Tissot.*) 2. In another, the same effect was produced by the slightest odor, even that of broth or of a medicine.—(*Schubart.*) 3. A child had a fit every time he saw something red.—(*Buchner, Tissot.*) 4. A child had an epileptic seizure as often as he heard a dog bark.—(*Van Swieten.*) 5. The idea of phantoms, which had frightened a boy, when quite young, was sufficient to cause the fits.—(*Maisonneuve.*) 6. In a case, the remembrance of a fright was enough to produce the seizure.—(*Van Swieten.*) 7. Any word of blame, addressed to two patients, gave them a fit.—(*Delasiauve.*)

The cases in which a physical impression has been the cause of the first attack of epilepsy, may be regarded as less valuable against the humoral hypothesis, than the preceding facts in which, at each return of the cause (either moral or physical,) a seizure took place. It might be said to diminish their value that the physical impression occurred just at the time when the poison of the blood was beginning to act upon the brain. But in admitting that such a coincidence has sometimes taken place, we certainly cannot imagine that *in all the very numerous cases of epilepsy*, in which the first fit has occurred immediately after a physical impression, such a coincidence has existed. The works of the principal writers on epilepsy, Van Swieten, Tissot, Maisonneuve, Cooke, Esquirol, Portal, Copland, Herpin, Delasiauve, Moreau, (de Tours), &c., contain too many of such facts for our dreaming of the possibility of explaining the production of epileptic fits, immediately after a physical impression, without attributing at least a share in the causation of these fits, to this impression. The *post hoc, propter hoc*, is a sound reasoning when the number of facts is so extremely considerable as it is here.

In my animals, as I have already said many times, the fits are produced at every time the skin of certain parts of the neck and face is pinched.* As the seizure in these animals takes place when we desire it, we have there a decided proof that, at least in them, fits may be produced otherwise than by the irritation of a poison on the encephalon.

It results from this exposition of facts that, in animals and in man, fits of epilepsy cannot be considered as always due to the influence of a poisonous matter upon the encephalon. We would not say, however, that they are never caused by a poison in the blood. It seems, on the contrary, not only when there is a deficiency in the urinary secretion, but also when the elements of bile are in great quantity in the blood, or when the functions of the supra-renal capsules are suppressed, that epileptiform seizures take place, owing to the irritation that certain substances, contained in the blood, exert upon some parts of the nervous system. When there is not a free menstruation, and perhaps, also, when the secretion of the skin is stopped, it seems probable that a poisonous matter remains in the blood, where it accumulates, and that it participates in the causation of epileptic fits.* Besides, it is certain that some poisons, and particularly lead, are able to cause epilepsy. But many questions are still to be solved, concerning the *modus operandi* of poisons which cause convulsions. I have shown elsewhere (*Experimental Researches applied to Physiol. and*

* In October, 1855, I had the satisfaction of showing this experiment to Dr. R. B. Todd himself, in presence of many distinguished physicians, among whom were W. Bowman, Prof. L. Beale, Dr. R. H. Semple, and Dr. R. Druitt.

* Very judicious remarks on the subject of the influence of poisonous matter contained in blood, in eruptive diseases, in jaundice, in deranged menstruation, in albuminuria, &c., have been made by Prof. Gunning S. Bedford, in his important work, *Clinical Lectures on the Diseases of Women and Children.* Third Ed. 1856. pp. 437, 475, 502, and 525–34.

*Pathol.* New York, 1853, p. 57–63 and p. 113) that these poisons have two modes of action, entirely different one from the other. One of these modes, which is by far the most frequent, seems to consist only in an increase of the reflex faculty of the cerebro-spinal centre. The poisons which belong to this category, according to my researches, are the following: strychnine, brucine, cyanhydric acid, cyanide of mercury, morphine, nicotine, picrotoxine, digitaline, sulphide of carbon, oxalic acid, &c. The other mode of action of poisons producing convulsions, consists mostly in a direct irritation of various parts of the nervous system. I do not know of any other poison, acting exclusively in this way, except a substance existing normally in the blood, which accumulates during asphyxia, and which very likely is carbonic acid. The differences between these two modes of action of poisons are striking. In one of these modes there is no irritation, or at least very little, produced upon the nervous system or the contractile tissues, and therefore there is no convulsion directly caused by the poisons belonging to this category.

It will probably surprise many persons to hear that strychnine, cyanhydric acid, brucine, &c., do not directly give convulsions—but this is a fact; these substances do not seem to have any power of excitation either on muscles, on sensitive and motor nerves, or even on the spinal cord. Perhaps some of the poisons, of which a list is to be found above, have a slight power of excitation on the spinal cord, but they certainly do not cause directly the powerful convulsions which are attributed to them. They act almost only in increasing the reflex power of the cerebro-spinal centre, in such a manner that the least excitation, as, for instance, a voluntary or a respiratory movement, or any other kind of irritation of nerves of the skin or of the mucous membranes, causes convulsive reflex movements. We might say that they act in giving to the nervous centres the faculty of causing convulsions *when the centres are irritated, but they do not irritate.* (For the proofs of these views, see my work above quoted, p. 57–63.) On the contrary, black blood, or very likely carbonic acid, seems to destroy the reflex power of the cerebro-spinal centre, but at the same time it *irritates* violently this centre, and, therefore, causes directly powerful convulsions. This last poison differs also from the preceding in being able to irritate directly muscles and motor or sensitive nerves. (See for this and other influences of black blood, or rather of carbonic acid, my work, already quoted, p. 110–13, and p. 117–24. See, also, the thesis of my friend and pupil, Dr. Brandt, entitled *Des phenomenes de contraction*

*observes chez des individus morts du cholera ou de la fievre jaune*, Paris, 1855, and my paper on red and black blood in the *London Medical Times and Gazette*, Nov. 17, 1855, p. 492–94.)

There are, therefore, some poisons that cause convulsions indirectly, by increasing the reflex power of the cerebro-spinal centre, and not in irritating them, while there are others which cause convulsions directly by an irritation of the cerebro-spinal centre. In which of these two categories are we to place the poisons, contained in blood in cases of epilepsy, where some secretion (the urinary, the biliary, &c.) is suppressed or much diminished? This is quite an undecided question. Many other things are still to be known concerning these poisons; but we do not intend to examine this subject here. We wished merely to say, that even in cases where there is some ground for the humoral theory of epilepsy, proposed by Dr. Todd, we have no proof that the poison acts as this eminent physician supposes. We will add that even in cases of organic disease of the kidney, coincident with epilepsy, we are not entitled to declare positively that it is in consequence of the accumulation of some of the principles of urine in the blood, that the fits are produced, as it might be that they result from an irritation of the renal nerves, as it is the case when there are calculi in the tubuli of the kidneys, without a notable diminution of the secretion of these glands. On another side it is very well known, as Prevost and Dumas, Segalas, Tiedemann and Gendrin, Mitscherlich, Bernard and Barreswil, Stannais, Frerichs and myself have ascertained many times, that after the extirpation of the kidneys, *i. e.*, when the urinary secretion is as much diminished as possible, convulsions are very rarely produced, and never violent. So that in a case of epilepsy with renal disease, either the convulsions have no relation whatever with the renal affection, or if they have a relation, it is either through the agency of the renal nerves, or in consequence of a transformation of some element of the urine in the blood, as these elements seem to be unable to cause convulsions. It is mostly this last argument which has led Frerichs, in his very interesting work on Bright's disease (*Die Bright'sche Nervenkrankheiten*, Leipzig, 1852), to his so-much-debated theory of uræmia.

As a general conclusion of our discussion of the humoral theory of epilepsy, we will say: 1st, that even in the cases where there is probably a poison in the blood, its relations with the production of fits is not known. 2d, that we are not entitled to consider as due to the elements of certain secretions, remaining in the blood, the epileptic fits which may exist when the glands producing these secretions are diseased. 3d, that there are a great many cases of epilepsy in which the cause of the fit is not in the blood.—*Boston Medical & Surgical Journal.*

